# In Vitro Comparison of the Efficacy of Peri-Implantitis Treatments on the Removal and Recolonization of *Streptococcus gordonii* Biofilm on Titanium Disks

**DOI:** 10.3390/ma11122484

**Published:** 2018-12-06

**Authors:** Selena Toma, Catherine Behets, Michel C. Brecx, Jerome F. Lasserre

**Affiliations:** 1 Department of Periodontology, Université Catholique de Louvain (UCL)—Cliniques Universitaires Saint Luc, 1200 Brussels, Belgium; michel.brecx@uclouvain.be (M.C.B.); jerome.lasserre@uclouvain.be (J.F.L.); 2 Institut de Recherche Expérimentale et Clinique (IREC), Pôle de Morphologie, Université Catholique de Louvain (UCL), 1200 Brussels, Belgium; catherine.behets@uclouvain.be

**Keywords:** peri-implantitis, biofilm, dental implants, in vitro model

## Abstract

**Objective:** To compare the efficacy of four commonly used clinical procedures in removing *Streptococcus gordonii* biofilms from titanium disks, and the recolonization of the treated surfaces. **Background:** Successful peri-implantitis treatment depends on the removal of the dental biofilm. Biofilm that forms after implant debridement may threaten the success of the treatment and the long-term stability of the implants. **Methods:** *S. gordonii* biofilms were grown on titanium disks for 48 h and removed using a plastic curette, air-abrasive device (Perio-Flow®), titanium brush (TiBrush®), or implantoplasty. The remaining biofilm and the recolonization of the treated disks were observed using scanning electron microscopy and quantified after staining with crystal violet. Surface roughness (Ra and Rz) was measured using a profilometer. **Results:** *S. gordonii* biofilm biomass was reduced after treatment with Perio-Flow®, TiBrush®, and implantoplasty (all *p* < 0.05), but not plastic curette (*p* > 0.05), compared to the control group. Recolonization of *S. gordonii* after treatment was lowest after Perio-Flow®, TiBrush®, and implantoplasty (all *p* < 0.05 vs. control), but there was no difference between the plastic curette and the control group (*p* > 0.05). Ra and Rz values ranged from 1–6 µm to 1–2 µm and did not differ statistically between the control, plastic curette, Perio-Flow, and TiBrush groups. However, the implantoplasty group showed a Ra value below 1 µm (*p* < 0.01, ANOVA, Tukey). **Conclusions:** Perio-Flow^®^, TiBrush^®^, and implantoplasty were more effective than the plastic curette at removing the *S. gordonii* biofilm and preventing recolonization. These results should influence the surgical management of peri-implantitis.

## 1. Introduction

Dental implants are a treatment option for the replacement of missing teeth, restoring dental function, and esthetics. However, approximately 30% of patients with implants develop peri-implantitis, a major reason for implant failure [1,2,3,4]. Peri-implantitis is characterized by biofilm-related inflammation of the tissues surrounding dental implants. The term peri-implantitis was first used in 1987 to describe a periodontitis-like disease characterized by biofilm-related inflammation of the tissues surrounding dental implants [5]. Subsequently, alveolar bone loss, visible by X-ray analysis, allowed peri-implantitis to be distinguished from peri-implant mucositis [6].

The colonization of the implant surface by oral bacteria organized in biofilms, similarly to periodontitis, is considered as a primary etiological factor of peri-implantitis [7]. Bacterial adhesion and biofilm organization in dental plaque play a crucial role in the pathogenesis of peri-implantitis [1]. Smoking and a history of periodontitis are risk factors for developing the disease [8,9]. Other risk factors have been highlighted recently, including excess cement, genetic polymorphisms, diabetes, cardiovascular diseases, and the absence of keratinized tissue adjacent to the implant [10].

As with periodontitis treatment, dental plaque removal is a major approach of peri-implantitis treatment. Numerous mechanical procedures have been proposed to remove the dental biofilm and to improve peri-implant health. From the least to the most abrasive techniques, these include using plastic, metals or ultrasonic scalers, rubber polisher, an air-powder abrasive device, rotating brushes, and implantoplasty protocols [11,12]. However, recent reviews have highlighted the absence of reliable evidence for the most effective interventions in peri-implantitis treatment owing to their complexity [13], and standardized, evidence-based protocols are still lacking [14]. For these reasons, a focus on the mechanical elimination of the dental biofilm instead of a combination of treatment modalities was preferred. Based on the instrumentation used for the treatment of periodontitis, the plastic curette appeared as a potential cleaning tool to remove dental biofilm from the titanium implant surface. The use of a non-metallic scaler was first advised to avoid major surface modification associated with a risk of lower biocompatibility and re-osseointegration. The studies evaluating the cleaning efficacy of an air-powder abrasive demonstrated constant results. According to Tastepe et al., in vitro cleaning efficiency of the method is reported to be high [15]. Airflow devices using glycine powders seem to constitute an efficient therapeutic option in the debridement of implants with peri-implantitis [16]. Rotating titanium brushes have been proposed as an alternative for the mechanical treatment of peri-implantitis. Meager results are reported in the literature. According to John et al., a rotating titanium brush seems effective for mechanical cleansing of sand-blasted, large grit, acid etched (SLA) surfaces, while inducing no surface alteration [17]. Implantoplasty consists of the elimination of surface roughness together with the implant threads. This technique has been proposed to optimize maintenance, and facilitate oral hygiene when implant threads are exposed. Few clinical studies have evaluated the effects of this protocol. Romeo and coworkers obtained an implant survival rate of 100% after three years, with improvement in clinical and radiological parameters, as compared to those without implantoplasty [18].

Early bacterial colonizers such as *Streptococcus gordonii* (*S. gordonii*) are known to play a crucial role for bacterial adhesion of middle colonizers (*Fusobacterium nucleatum*, *F. nucleatum*) and late colonizers (*Porphyromonas gingivalis*, *P. gingivalis*) in the beginning of the formation of oral biofilm [19]. Therefore, elimination of early colonizers on the surface of dental implants could be decisive for long term implant success.

Therefore, the present in vitro study aims to compare the efficacy of four mechanical methods (a plastic curette, an air-abrasive device (Perio-Flow), titanium brush (TiBrush), and implantoplasty) on the removal of *Streptococcus gordonii* from titanium disks, and the bacterial recolonization of disks previously treated.

## 2. Materials and Methods

**Titanium disks**

Two hundred and fifty sterile, microrough titanium disks (diameter, 5 mm; thickness, 2 mm) that had been sand-blasted with aluminum oxide beads (75–170 µm) and treated with solvents by the manufacturer (Southern Implants, Irene, South Africa) were used. The disks were handled by their circumference to avoid contact with the surface to be treated and analyzed.

### 2.1. Decontamination Assay

**Saliva coating of the disks**

As already described by Ota-Tsuzuki et al. [20], unstimulated saliva was collected from three healthy donors (aged 24–26 years) for 30 min per day for 7 days. After collection of 300 mL, the saliva samples were frozen at −20 °C. Then, the saliva samples were pooled and centrifuged (6000× *g* rpm for 30 min at 4 °C), and the supernatant was filtered (5 µm and 0.22 µm). The sterile disks were placed in a sterile 24-well polystyrene cell-culture plate containing 500 µL saliva per well for 30 min at 37 °C to allow salivary pellicle formation.

**Biofilm formation**

The disks were placed in a new 24-well polystyrene plate after aspiration of the saliva. Standard reference-strain *S. gordonii* (ATCC 10558) was used to prepare inoculum. Inoculation, and incubation was done under anaerobic conditions (80% N_2_, 10% H_2_, and 10% CO_2_) for 24 h at 37 °C. The bacterial cells were suspended in BHI agar, adjusting the turbidity to an optical density (OD) of 0.15 at 630 nm with 106 colony-forming units/mL, and 500 µL of this suspension added to the wells and incubated for 48 h under anaerobic conditions [20]. After formation of the *S. gordonii* biofilms, unattached cells were removed by washing with sterile saline solution, placed in a new sterile 24-well plate, and randomly allocated to the different treatment groups.

**Titanium surface treatment**

In all groups, experiments were carried out using 10 disks/group and performed five times (n = 5). As already described in Toma et al., [21], the disks were treated with plastic curette, Perio-Flow, titanium brush (Ti-Brush), and implantoplasty. Non-treated disks were used as controls.

**Plastic curette**—The entire surface of the disk was scaled with a plastic curette (*Implacare, Hu-Friedy, Chicago, IL, USA) at an angle of 70° for 30 s. The tip of the curette was made from high-grade resin. Each side of the curette was used for five disks.

**Perio-Flow**—The disks were treated using an air-abrasive system (^†^ Perio-Flow, Perio-Flow nozzle, EMS, Nyon, Switzerland) using tap water and an air-power setting with glycine powder (25 µm) (Air-Flow Perio Powder, EMS). The specially designed nozzle, consisting of a thin flexible plastic tube (length 1.7 cm; diameter 0.8 mm at the tip), was fixed on a handpiece (Air-Flow EL-308/A, EMS, Nyon, Switzerland). Perio-Flow was applied in a circular, non-contact mode, parallel to the disk surface for 30 s. After, glycine powder was removed by irrigation with sterile saline (20 mL, 20 s).

**Titanium brush**—The Ti-Brush^®^ (^‡^ Straumann^®^, Basel, Switzerland) is made of titanium bristles with a stainless steel shaft. Disks were processed using a Ti-Brush^®^ fixed on a surgical handpiece (^§^Bien-Air Medical Technologies, Bienne, Switzerland) oscillating in a clockwise/counterclockwise direction at low speed (maximum of 900 oscillations per minute, 30 s). Sterile saline solution (NaCl 0.9%) was used for irrigation and cooling of the treatment site. Each brush was used for five disks and then replaced.

**Implantoplasty**—Disks were polished with a diamond round shaped bur (30 µm particle size egg-shaped bur) (^‖^ Komet, Gerb. Brasseler GmbH, Lemgo, Germany) and assembled on a handpiece (^¶^KaVo Dental GmbH, Biberach, Germany) working at 15,000 rpm. The disks were treated for 30 s and rinsed with sterile saline to remove any titanium particles.

**Crystal violet assay**—Crystal violet was used to evaluate the total amount of biofilm. After treatment, the disks were dried at 45 °C for 60 min, then immersed in a 1% crystal violet solution in the dark, at room temperature for 15 min. After three rinses in phosphate-buffered saline, 1 mL acetic acid (33%) was added to each well and left in the dark at room temperature for 15 min. The absorbance was measured at 450 nm using a microplate spectrophotometer (^#^Bio-Rad Laboratories, CA, USA) and reported as OD.

### 2.2. Recolonization Assay

Treated disks (10/group, repeated five times) were assigned to their experimental groups, coated with saliva, and cultured with *S. gordonii* to obtain a biofilm, as described above. This biofilm was also assessed using crystal violet staining and OD measurement after 48 h.

**Scanning electron microscopy (SEM)**

SEM was used to examine the remaining *S. gordonii* biofilm (48 h culture, n = 3/group, in duplicate) after each of the four treatments, as well as the biofilm recolonization of previously treated titanium disks. Bacterial biofilm was fixed in 2.5% glutaraldehyde in 0.05 mol at pH 7.4 for 1 h, post-fixed with 1% osmium tetroxide at pH 7.4 for 1 h, and dehydrated through an ethanol series (30%, 50%, 70%, 90%, and 100%; 20 min per concentration). Finally, the disks were sputter-coated with gold (^††^ Emitech K550; Houston, TX, USA) and examined under a scanning electron microscope (JEOL 7200, Tokyo, Japan) at 15 kV.

**Measurement of surface roughness**

The surface roughness of the control and treatment groups was measured using a contact profilometer (DektakXT Bruker Stylus Profiler; Billerica, MA, USA) equipped with a diamond microneedle (diameter, 7 µm). The profilometer scanned each disk along a length of 2 mm. The horizontal movements of the tip, generated by surface irregularities, were transferred to a transducer that created an electric stimulus. Three measurements per disk (in duplicate) were taken. All measurements were carried out in the same direction. Arithmetical mean roughness (Ra) and ten-point mean roughness (Rz) were recorded.

**Statistical analysis**

Data are expressed as mean ± SD. To compare data between the groups, those following a normal distribution and homogeneity of variance were analyzed using a one-way analysis of variance (ANOVA) and a Tukey *post-hoc* test. Those following a non-normal distribution were analyzed using a non-parametric Kruskall–Wallis test with a Nemenyi–Damico–Wolfe–Dunn post-hoc test (GraphPad InStat version 3; GraphPad Software, La Jolla, CA, USA). Differences were considered statistically significant when *p* < 0.05.

## 3. Results

### 3.1. Bacterial Elimination After Surface Decontamination

**Figure 1:**
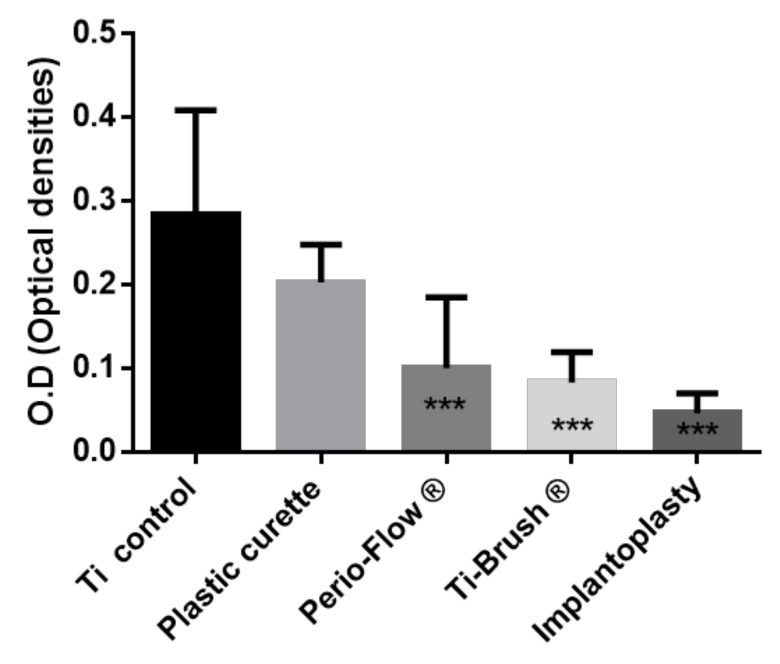
*Streptococcus Gordonii* Biofilm elimination after treatment. Results expressed in optical densities (O.D) (Crystal violet staining). Ti control vs. Plastic curette (ns *p* > 0.05), Ti control vs. Perio-Flow ® ** *p* <0.001, Ti control vs. Ti-Brush ® ** *p* < 0.001, Ti control vs. Implantoplasty *** *p* < 0.001, Plastic curette vs. Perio-Flow ® * *p* < 0.05, Plastic curette vs. Ti-Brush ® *** *p* < 0.001,Plastic curette vs. Implantoplasty *** *p* < 0.001, Perio-Flow ® vs. Ti-Brush ® ns *p* > 0.05,Perio-Flow ® vs. Implantoplasty ns *p* > 0.05, Ti-Brush ® vs. Implantoplasty ns *p* > 0.05 *(Kruskal Wallis, Dunn’s).*

Decontamination of the titanium surfaces by the different procedures was quantified by measuring the in vitro biofilm biomass using OD after crystal violet staining and SEM. Biofilm OD was significantly lower in the Perio-Flow^®^, TiBrush^®^, and implantoplasty groups than in the control and plastic curette groups (*p* < 0.05), demonstrating elimination of a greater part of the biofilm (Figure 1). No statistical difference was observed between the biofilm biomass of the control and plastic curette groups (*p* > 0.05).

**Figure 2:**
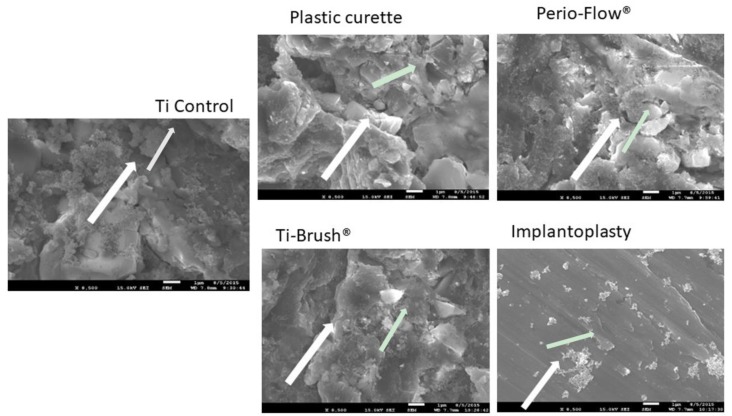
Scanning electron micrographs of Streptococcus Gordonii biofilm (48 h) elimination on titanium disks after treatments (8500 ×). Biofilm colonies were more abundant on the untreated disks, the plastic curette group than on other disks. Few bacteria were visible on the implantoplasty-treated disks, allowing the smooth titanium surface to be observed.

SEM revealed that biofilm colonies were more abundant on the untreated disks and those treated with the plastic curette than on other disks (Figure 2). The metallic surface of the disks was hardly visible in the control, plastic curette, Perio-Flow^®^, and TiBrush^®^ groups, as they were covered in biofilm, whereas few bacteria were visible on the implantoplasty-treated disks, allowing the smooth titanium surface to be observed.

### 3.2. Bacterial Recolonization After Surface Treatment

**Figure 3:**
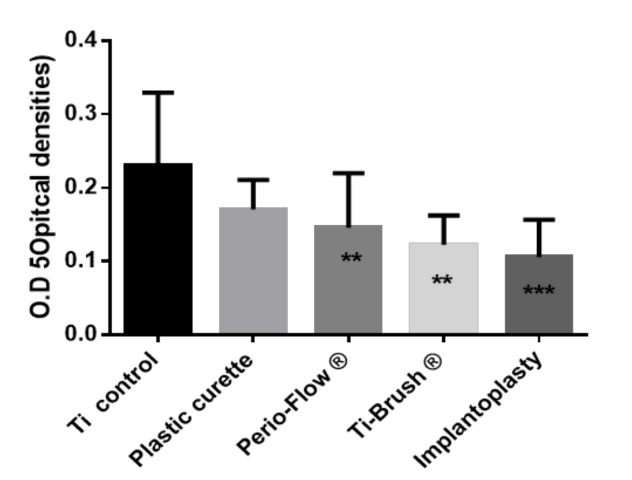
*Streptococcus Gordonii* biofilm recolonisation on treated disks. Results expressed in optical densities (O.D) after Crystal violet staining. Ti control vs. Plastic curette ns *p* > 0.05, Ti control vs. Perio-Flow ® ** *p* < 0.01, Ti control vs. Ti-Brush ® *** *p* < 0.001, Ti control vs. Implantoplasty * *p* < 0.05, Plastic curette vs. Implantoplasty *** *p* < 0.001, Perio-Flow ® vs. Ti-Brush ®ns *p* > 0.05, Perio-Flow ® vs. Implantoplasty ns *p* > 0.05, Ti-Brush ® vs. Implantoplasty ns *p* > 0.05 *(Kruskal Wallis, Dunn’s*).

**Figure 4:**
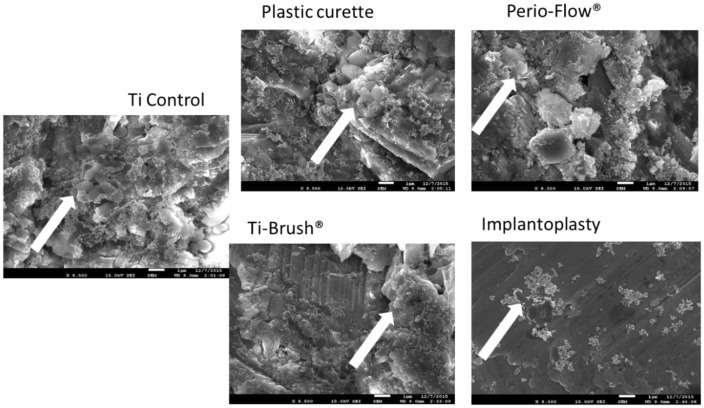
Scanning electron micrographs of Streptococcus Gordonii biofilm colonization (48 h) of previously treated titanium disks (8500 ×). A multilayered *S. gordonii* biofilm was present on all titanium surfaces, with a more pronounced colonization of the control and plastic curette groups. Few bacteria were visible on the implantoplasty-treated disks, allowing the smooth titanium surface to be observed.

The OD of the *S. gordonii* bacterial biomass was quantified after 48 h of culture and crystal violet staining of previously treated titanium disks (Figure 3). OD was significantly lower on the disks treated with Perio-Flow^®^, TiBrush^®^, and implantoplasty than on the control disks (*p* < 0.05). There was no difference in OD between the control and plastic curette groups (*p* > 0.05). SEM revealed a multilayered *S. gordonii* biofilm on all titanium surfaces, with more pronounced colonization of the control and plastic curette groups and more titanium visible on the implantoplasty group (Figure 4).

### 3.3. Surface Roughness

All descriptive data for Ra and Rz is presented in Table 1. The surface of the implantoplasty group was smoother (Ra < 1 µm) than that of the control group (*p* < 0.01, ANOVA, Tukey). There was no statistically significant difference between the control, plastic curette, Perio-Flow, and TiBrush groups (*p* > 0.05, ANOVA, Tukey).

## 4. Discussion

The aim of the present study was to compare the efficacy of four mechanical methods—plastic curette, an air abrasive device (Perio-Flow^®^), TiBrush^®^, and implantoplasty—in decontaminating and preventing the recolonization of titanium.

Within the limitations of this in vitro study, the plastic curette was the least effective method for removing *S. gordonii*. In contrast, the Perio-Flow^®^, TiBrush^®^, and implantoplasty methods eliminated significantly more of the biofilm. This study also showed that biofilm growth was significantly lower on the disks treated using Perio-Flow^®^, TiBrush^®^, and implantoplasty than on the untreated disks and those treated using the plastic curette.

If preservation of surface integrity is the primary therapeutic objective, the plastic curette may be preferable in the case of mucositis; nevertheless, its ability to effectively remove calculus and biofilm from smooth and rough surfaces has been widely questioned [22]. Previous studies showed no marked alterations of smooth or rough surfaces after treatment with plastic curettes [22,23,24]. The results obtained in the present study are consistent with this and suggest that this technique should not be considered as a treatment for peri-implantitis.

Air-powder abrasive systems were introduced to decontaminate implant surfaces following the failure of non-metallic instruments. Glycine powder, which does not markedly change implant surfaces or damage fibroblast attachment, is recommended for the treatment of rough implant surfaces [12,22]. Light titanium surface changes, such as rounding of the angles and edges of rough surfaces and occasional surface pitting, are visible using SEM after treatment with the air-powder system [25]. In contrast, no quantifiable change in roughness is detected on smooth or rough surfaces using a profilometer [26], which is in agreement with present results. It is important to note that surface changes may vary according to titanium hardness, duration of exposure, air pressure, size and hardness of the abrasive particles, and distance and angulation of the tip [27]. According to Menini et al., air polishing using either glycine or sodium bicarbonate powder for 5 or 20 s of machined and acid-etched titanium surfaces does not damage surface morphology [28].

In this study, TiBrush^®^ treatment produced no significant changes in Ra on the titanium surface, although slight changes were observed in the SEM images (Figure 2) as previously demonstrated [21]. Ra averages all peaks and valleys of the roughness profile and thus could be considered too general. Nevertheless, it remains one of the most widely used parameters of roughness and is considered a good general indicator of the surface texture [29,30]. In accordance with previous results, TiBrush^®^ seemed to be an effective instrument for mechanical cleansing while being gentle to the implant surface [17]. Combining this technique with a non-mechanical treatment such as photodynamic therapy increases its effectiveness on smooth and SLA surfaces [31].

Implantoplasty has been increasingly studied over the past decade [18,32,33,34] and now appears to be a viable alternative treatment for controlling peri-implantitis. The aim of this method is to polish the implant surface, then to decontaminate the implant surface, and finally to create a new surface less prone to plaque and bacterial adhesion [35]. The data obtained in the present study confirm that this method reduces surface roughness (Ra). However, the Ra we obtained is high compared to the results from a previous study [35]. This could be explained by the fact that we used only one diamond bur in this in vitro study. It seems that a combination of different burs associated with a polishing stone is recommended in order to reduce the Ra value considerably [35]. A recent study validated this technique for the management of exposed SLA implant surfaces regarding biocompatibility [36].

Instruments used to decontaminate implants should also attempt to reduce *de novo* bacterial recolonization and biofilm formation, as previously described by Duarte et al. [26]. In the present study, titanium disks treated with Perio-Flow^®^, TiBrush^®^, and implantoplasty demonstrated less *S. gordonii* adhesion than untreated control surfaces and plastic curette-treated surfaces, probably due to the difference of surface profiles observed using SEM (Figure 4). Indeed, the texture produced by TiBrush^®^ and implantoplasty is characterized by flattened edges and a smoother surface (Figure 4). The low bacterial adhesion on the surfaces treated with the air-powder abrasive system could be explained by the presence of deposits of glycine powder. Moreover, our results suggest that the type of instrument plays an important role in *de novo* biofilm formation on rough surfaces.

Surface roughness is considered one of the most important factors influencing oral biofilm formation [37]. Rougher surfaces are conducive to bacterial adhesion [37,38], plaque formation, and plaque adherence [39,40,41]. According to Quirynen et al., roughness provides a large surface area and niches for microbial adhesion, reduces shear forces, and reduces bacterial desorption during initial adhesion [42]. Free surface energy determines biofilm development and plaque formation. The contact angle of each material influences surface energy. In the present study, surface roughness appeared to be associated with low bacterial adhesion. In previously published data, implantoplasty-treated titanium disks appeared smoother than control disks [21]. The difference in bacterial elimination and adhesion between the plastic curette and implantoplasty groups could be explained by this change in surface topography.

Furthermore, recent data confirmed the hydrophilic character of the implantoplasty-treated titanium disks [21]. Bacterial adhesion is promoted on hydrophobic surfaces via proteins acting as specific binding sites for bacteria. Bacterial adhesion is thus precipitate and facilitate [43]. Hydrophilization of surfaces has been shown to inhibit biofilm development [44] and could explain the low adhesion of bacteria on implantoplasty-treated disks. This method, performed during surgical debridement, could become the preferred treatment for peri-implantitis [45]. The modification of wettability observed in the implantoplasty group, in association with its smooth aspect and its particular chemical composition, may represent an advantage in terms of biocompatibility [21].

One limitation of this study is the fact that the oral cavity environment cannot be identically reproduced due to its mixed microbiota, shearing forces, and the antimicrobial effects of the saliva [46]. Biofilms formed in vivo and in vitro are not easily comparable [46]. In vivo, questions remain on the role of the salivary acquired pellicle on bacterial adhesion [47]. Another limitation is the fact that we investigated the effect of treatments on the decontamination and adhesion of only one bacterial species (*S. gordonii*). Although they are associated with healthy implant sites, streptococci—particularly *S. gordonii*—are considered early colonizers and accessory pathogens that facilitate the attachment of organisms normally incapable of binding to host surfaces and, therefore, can lead to biofilm development [48]. Many secondary colonizers, adherent to early colonizers previously present in the biofilm, are known to be implicated in peri-implant diseases like *Fusobacterium, Capnocytophaga, Porphyromonas*, and *Prevotella spp.* [49]. The use of this monospecies *S. gordonii* biofilm allowed us to reproduce early surface contamination. Furthermore, *Streptococcus gordonii* cells are known to be hydrophobic. Adhesion of microorganisms on different types of surfaces can be influenced by their hydrophobicity [49]. Hydrophobic surfaces attract more hydrophobic cells, while hydrophilic surfaces are more attractive for hydrophilic cells [50,51]. Since implants are made of hydrophobic materials, hydrophobic microorganisms easily adhere to them. The use of another bacterial species may have led to different results. Furthermore, a longer culture time (> 48 h) (more similar to what happens in vivo) might lead to more pronounced differences between groups. Roughness could emerge as a disadvantage as it renders the surface more prone to bacterial adhesion [37].

A decrease in O.D values can be observed between decontamination and recolonization (Figure 1 and Figure 3) in the control group and plastic curette group, explaining this difference.

In this study, titanium disks were used instead of entire dental implants. The macrostructure of entire dental implants differs considerably from that of titanium disks, but the disks have been used in several previous in vitro studies to test experimental conditions [52,53]. The configuration of the peri-implant lesion, as well as implant surface accessibility, should also be considered prior to treating peri-implantitis. Whenever possible, pre-operative removal of the superstructure is recommended to ensure accessibility. Because of these morphological particularities, large rotary instruments, such as an implantoplasty diamond bur, might not be appropriate in some clinical situations.

## 5. Conclusions

This study showed that Perio-Flow^®^, TiBrush^®^, and implantoplasty protocols were more efficient than the plastic curette to remove in vitro *S. gordonii* biofilm from titanium disks. The effects of implantoplasty on the surface properties of the disks highlight it as a promising treatment for peri-implantitis. Further ex vivo microbiological studies should be performed to confirm these results.

## Figures and Tables

**Table 1:** The surface roughness of the control group and the four treatment groups were measured using a contact profilometer (DektakXT, Bruker, Billerica, MA, USA). Ra and Rz are expressed in µm. Three measurements per disks (in duplicate), repeated twice, were performed in parallel directions (Anova, Tukeys, ns *p* > 0.05).

Treatment	Ra (Mean ± SD)	Rz (Mean ± SD)	*p* Value
Control group	1.65 ± 0.107	9.79 ± 0.34	ns
Plastic curette	1.61 ± 0.17	9.98 ± 0.24	ns
Perio-Flow^®^	1.31 ± 0.14	9.22 ± 0.27	ns
Ti-Brush^®^	1.22 ± 0.31	8.76 ± 0.15	ns
Implantoplasty	0.98 ± 0.12	7.26 ± 0.022	** *p* < 0.01
